# Apical Groove Type and Molecular Phylogeny Suggests Reclassification of *Cochlodinium geminatum* as *Polykrikos geminatum*

**DOI:** 10.1371/journal.pone.0071346

**Published:** 2013-08-19

**Authors:** Dajun Qiu, Liangmin Huang, Sheng Liu, Huan Zhang, Senjie Lin

**Affiliations:** 1 CAS Key Laboratory of Tropical Marine Bio-resources and Ecology, South China Sea Institute of Oceanology, Chinese Academy of Science, Guangzhou, China; 2 Department of Marine Sciences, University of Connecticut, Groton, Connecticut, United States of America; American University in Cairo, Egypt

## Abstract

Traditionally *Cocholodinium* and *Gymnodinium* sensu lato clade are distinguished based on the cingulum turn number, which has been increasingly recognized to be inadequate for Gymnodiniales genus classification. This has been improved by the combination of the apical groove characteristics and molecular phylogeny, which has led to the erection of several new genera (*Takayama, Akashiwo*, *Karenia*, and *Karlodinium*). Taking the apical groove characteristics and molecular phylogeny combined approach, we reexamined the historically taxonomically uncertain species *Cochlodinium geminatum* that formed massive blooms in Pearl River Estuary, China, in recent years. Samples were collected from a bloom in 2011 for morphological, characteristic pigment, and molecular analyses. We found that the cingulum in this species wraps around the cell body about 1.2 turns on average but can appear under the light microscopy to be >1.5 turns after the cells have been preserved. The shape of its apical groove, however, was stably an open-ended anticlockwise loop of kidney bean shape, similar to that of *Polykrikos*. Furthermore, the molecular phylogenetic analysis using 18S rRNA-ITS-28S rRNA gene cistron we obtained in this study also consistently placed this species closest to *Polykrikos* within the *Gymnodinium* sensu stricto clade and set it far separated from the clade of *Cochlodinium*. These results suggest that this species should be transferred to *Polykrikos* as *Polykrikos geminatum*. Our results reiterate the need to use the combination of apical groove morphology and molecular phylogeny for the classification of species within the genus of *Cochlodinium* and other Gymnodiniales lineages.

## Introduction

Gymnodiniales is a major order of dinoflagellates, which are ecologically important because many of its species, such as *Cochlodinium polykrikoides, Karenia mikimotoi,* cause harmful algal blooms. While their correct classification is crucial for understanding their varying ecological characteristics, the traditional genus delimitation criteria in the unarmoured order of dinoflagellates have proven to be problematic [1], [2]. Many genera were described during the 1800s or early 1900s, and the taxonomy is sometimes artificial and misleading. For instance, *Gymnodinium* Stein, *Gyrodinium* Kofoid and Swezy, *Amphidinium*, and *Katodinium* Fott were defined on the basis of the relative sizes of the epicone and hypocone [1], which is not reliable. Genus delimitations can be further obscured by similar morphological characteristiccharacteristics shared between two or more genera [2]. Since the beginning of this century, some researchers proposed alternative classification schemes to address some of the apparent taxonomic problems and to improve our understanding of the phylogeny within the order [1]–. As a result, some genera (e.g., *Gymnodinium* Stein, *Gyrodinium* Kofoid and Swezy, and *Amphidinium* Claparede et Lachmann) have been redefined [1], [4], [7]–[8]. Furthermore, several new genera have been erected (*Akashiwo*, *Karenia*, *Karlodinium*, *Takayama*) and moved out of the initial *Gymnodinium* genus (sensu lato) based on molecular phylogeny and the characteristics of the apical groove. The combination of apical groove morphological and molecular phylogenetic studies has led to the uncovering of many relationships that were previously unknown [1]–[11]. However, some genera, such as the genus of *Cochlodinium,* are still defined based on the old genus discriminating criteria [12], [13]. It is necessary to re-examine species in *Cochlodinium* based on the combination of apical groove morphology and molecular phylogeny.

*C. geminatum* presents an excellent case calling for the re-examination of *Cochlodinium* taxonomy. This species was first described as *Gymnodinium geminatum* with its cingulum reported to wrap around the cell body 1.5 or 2.0 turns [13], [14]. After Schütt (1896) defined cingulum surrounding the cell at least 1.5 turns as the delimitating criterion of the genus of *Cochlodinium*, it was transferred to the genus *Cochlodinium*. It was reported in River Derwent, Triabunna, Australia in 2010 [15] and has been found to cause blooms in Pear River Estuary, China in recent years [16], [17]. Unlike *C. polykrikoides*, which caused mass mortality of fish while forming blooms, *C. geminatum* has not inflicted such ecological devastation even in its massive blooms in Pearl River Estuary, China. It is important to ascertain the taxonomic relationship between *C. geminatum* and *C. polykrikoides* for understanding their different ecological behaviors. In this study, we analyzed the ultrastructure, the major accessory pigment, and multi-gene phylogeny of the population to determine its relationship with other species in the *Cochlodinium* genus and other Gymnodiniales lineages. Our results clearly indicated that *C. geminatum* should be placed in the genus *Polykrikos*, reiterating that the traditional cingulum turn number is unreliable and should be replaced by apical groove morphotyping combined with molecular phylotyping for the classification of *Cochlodinium* and related species.

## Materials and Methods

### Sampling from the bloom event and isolation of *C. geminatum* cells

Water samples were collected on August 25, 2011 at 22°9.8′N, 113°37.5′E near Zhuhai in the Pearl River Estuary, South China Sea, during a bloom event that lasted for >7 days. No specific permissions were required for sampling at this location. Meanwhile, our field studies did not involve endangered or protected species. One subsample was transferred into a 500-mL clean plastic container and preserved with Utermöhl's solution [18]. The sample was then stored in the dark at room temperature until microscopic morphological analysis (within 2 months). Six 2-mL subsamples were preserved in 2% formaldehyde and immediately frozen in liquid nitrogen and stored at −80°C for microscopic pigment fluorescence examination. Besides, a 50-mL water sample was filtered onto a 20-µm membrane and preserved in liquid nitrogen for subsequent HPLC analysis of pigment composition. In addition, a live sample was brought back to the laboratory in Guangzhou for microscopic observation of the mode of cell movement. In the laboratory, from the original Utermöhl's-fixed samples, twenty *C. geminatum* cells were isolated under an inverted microscope. The isolated cells were rinsed carefully with 0.45 µm-filtered seawater and used for subsequent DNA extraction.

### Culture of North American *C. polykrikoides* and sample collection

A culture of *C. polykrikoides* originally isolated from Gulf of Maine (kindly provided by Don Anderson, WHOI) was grown in L1 medium [19] at 25°C under a 14:10 L:D cycle with a photon flux of 125±20 mE s^−1^ m^−2^. Cells were harvested using centrifugation at 3000×g for 10 min at 4°C. The cell pellet was immediately stored at −80°C for subsequent DNA analysis.

### Light microscopy (LM) and cell sorting

Cells of *C. geminatum* fixed in formaldehyde and Utermöhl's solution were observed under an Olympus BX51 epifluorescence microscope. Morphological identification of the species was done following Kofoid and Swezy (1921) and Schütt (1895, 1896) [12], [13], [14]. A subsample of the formaldehyde-fixed sample was incubated in SYBR Green I solution (35149A, Molecular probes, Invitrogen Corporation, Carlsbad, CA, USA, with 10^4^ –fold dilution) at room temperature in the dark for ten minutes to stain DNA in the nucleus of the cell. The stained samples were examined under the epifluorescence microscope [20].

### Scanning electron microscopy (SEM)

The Utermöhl's-fixed cells were allowed to settle by gravity, the supernatant decanted, and 1 ml of the 5% buffered glutaraldehyde fixative was added to the pellet as a post fixative. After 4 h at 4°C, the glutaraldehyde-fixed cells were allowed to settle by gravity again, and washed three times in 0.1 M phosphate buffer solution (PBS, pH7.2). Then 0.5 ml of 2% osmium tetroxide solution in the cacodylate buffer was added to the pellet as a post stain and fixative. After 12 h at 4°C, 0.5 ml of 2% osmium-fixed cells were allowed to settle by gravity again, washed three times by PBS and dehydrated in a graded ethanol /aqueous series (30%, 50%, 70%, 85%, 95% and 100% [twice] for 20 min each), acetone and isoamyl acetate (100%, twice for 20 min each). The cells were critical point-dried with CO_2_ using a Tousimis Samdri 795CPD (Rockville, MD, USA). Dried coverslips were amounted to aluminum stabs and then sputter coated with gold using a Denton Desktop 2 sputter coater. The coated cells were observed with a Hitachi S-3400N scanning electron microscope (Hitachi, Japan).

### Transmission electron microscopy (TEM)

Ten ml of the samples fixed in 2% formaldehyde (final concentration) and stored −80°C were used for TEM analysis. The samples were centrifuged at 6000×g for 10 min and the pelleted cells were resuspended in 2 mL of 5% buffered glutaraldehyde fixative and allowed to fix at 4°C for 48-hours. The re-fixed cells were gently centrifuged at 6000×g for 10 minutes and resuspended in 0.5 mL of 2% osmium tetraoxide solution prepared in the cacodylate buffer as a post stain and fixative. After 12 h at 4°C, the osmium-fixed cells were sedimented by gravity, washed in PBS buffer, dehydrated in a graded ethanol/aqueous series and acetone, and then imbedded in SciPon resin in beam capsules at 70°C for 18 h. Ultrathin sections, collected on uncoated copper grids, were obtained with a Porter-Blum MT-2 ultramicrotome fitted with a diamond knife, post-stained with Reynold's lead citrate, and observed under a JEM-100CXII transmission electron microscope (Jeol Inc., Tokyo, Japan).

### Pigments analysis by high performance liquid chromatography (HPLC)

The 50-mL blooms water sample was gently filtered onto 25-mm diameter 20 µm-pore size fiber filters and immediately stored in liquid nitrogen. Filters were subsequently transferred to 2.5 mL of methanol, sonicated for 30 s, and filtered (0.2 µm pore size, Millipore). One milliliter of this filtrate was mixed with 250 µL of distilled deionized water immediately before analysis. HPLC analyses were performed on a Shimadzu LC 10A system with a Supelcosil C18 column (250×4.6 mm, 5 µm) using the method of Wright et al. (1991) [21]. The types of major pigments were identified by typical retention times and absorption spectra of the separated peak fractions for dinoflagellates.

### DNA extraction, PCR amplification and sequencing of nuclear and mitochondrial genes

Twenty *C. geminatum* cells and a cell pellet containing ∼1000 cells of *C. polykrikoides* were put into separate 1.5 mL microtubes and re-suspended in 0.5 mL DNA lysis buffer (0.1M EDTA pH8.0, 1% SDS, 200 μg mL^−1^ proteinase K) and incubated for 48 hours at 55°C. DNA extraction of both samples followed a previously reported CTAB protocol [22] and further purified using Zymo DNA Clean and Concentrator kit (Zymo Research Corp., Orange, CA). The final DNA elution step in DNA Clean procedure was done using 20 µL of 10 mM Tris-HCl solution (pH 8.0).

Using 1 µL of the extracted DNA as the template, PCR was carried out to amplify fragment of ribosomal RNA (rDNA) gene cluster containing the majority of the small subunit rDNA (18S), complete internal transcribed spacers (ITS) 1, 5.8S rDNA, and ITS2 (ITS1-5.8S-ITS2), and a 5′end fragment of the large subunit rDNA (28S). Several pairs of primers were used amplify each of these rDNA regions (Table 1), [23]–[26]. PCR was performed under the following condition: one initial denature step at 94°C for 3 min followed by 35 cycles of 94°C for 30 sec, 56°C for 30 sec, and 72°C for 45 sec, finalized by 10 min at 72°C for one cycle. PCR products were resolved by agarose gel electrophoresis and the bands with expected sizes were excised. DNA was purified and directly sequenced as reported [20].

**Table 1:** Primers used in the present study.

Primer name	Annealing temps	Application	Sequence (5′–3′)	References
Dino18SF1	56°C	PCR for 18S	AAGGGTTGTGTTYATTAGNTACARAAC	[23]
18ScomR1			CACCTACGGAAACCTTGTTACGAC	[24]
Dino1662 F	56°C	PCR for 18S-28S long fragment	CCGATTGAGTGWTCCGGTGAATAA	[25]
28S R2			ATTCGGCAGGTGAGTTGTTAC	[26]

### Alignment and phylogenetic analyses

The DNA sequences obtained were used to search against GenBank databases using Basic Local Search Tool (BLAST) to determine what organisms these sequences represented. Thirty to 50 sequences showing significant similarity in BLAST to the sequences obtained in this study were retrieved from the databases. Phylogenies based on partial 18S, partial 28S (D1–D4, 1, 264 bp) and the concatenation of these two (18S+28S) were used to investigate the phylogenetic position of *C. geminatum*. These datasets were separately aligned using ClustalX. The alignments were run through ModelTest to select the most appropriate evolutionary model. The selected General Time Reversible (GTR) model with gamma distribution was employed for Maximum Likelihood analysis using PhyML3.0 aLRT [27]. Categories of substitution rates were set at 4, and other parameters were estimated based on the datasets. The proportion of invariable sites and gamma shape parameter were 0.267 and 0.514, respectively for the 18S dataset, 0.000 and 0.436 for 28S, 0.185 and 0.345 for 18S+28S.

## Results

### Morphological characters

**Figure 1:**
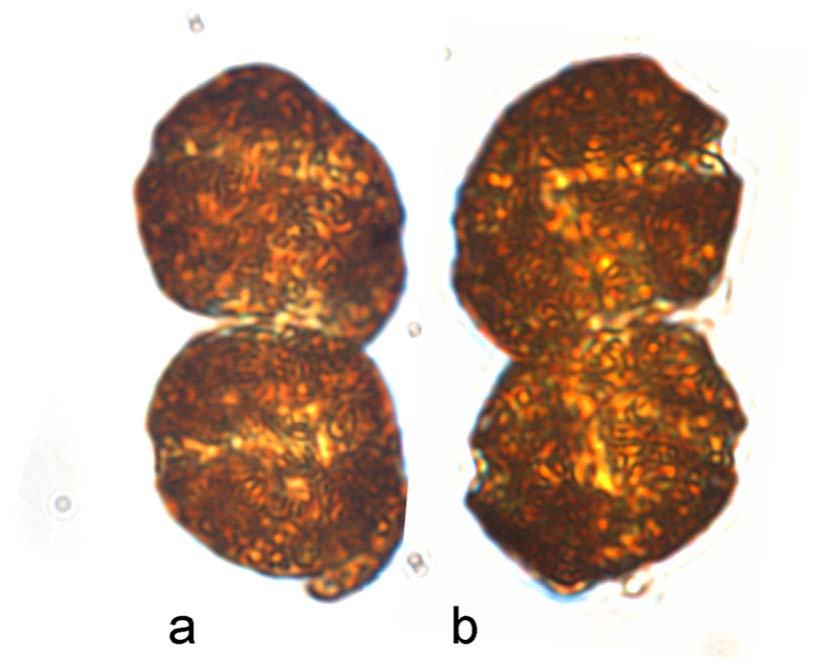
Light microscopic photographs of *P. geminatum* ( = *C. geminatum*) paired cells preserved in different fixatives. a–b) Side view of cells fixed using Utermöhl's. Note that Utermöhl's fixation effectively preserved cell morphology, with visible cingulum and sulcus.

**Figure 2:**
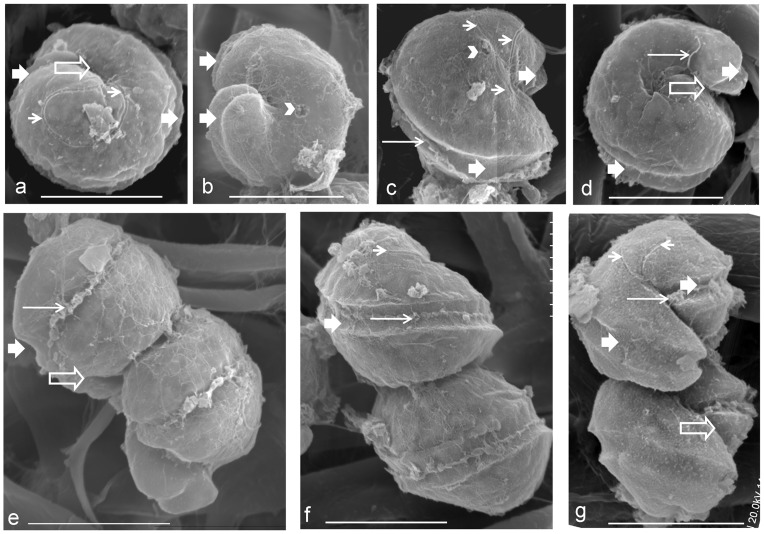
Scanning electron microscopic (SEM) photographs of the paired cells of *P. geminatum* ( = *C. geminatum*) to show the apical groove (short thin arrow), connection pore (arrow head, in panel b), cingulum (short thick arrow), flagellum (long thin arrow), and sulcus (long hollow arrow) (a–g). a) Apical views of the epitheca in the upper cell. b) Antapical views of the hypotheca in the upper cell. c) Vertical views of the lower cell. d) Antapical views of the hypotheca in the lower cell. e–g) Oblique ventral views of dual cells. Scale bar in all the photographs  = 20 µm.

**Figure 3:**
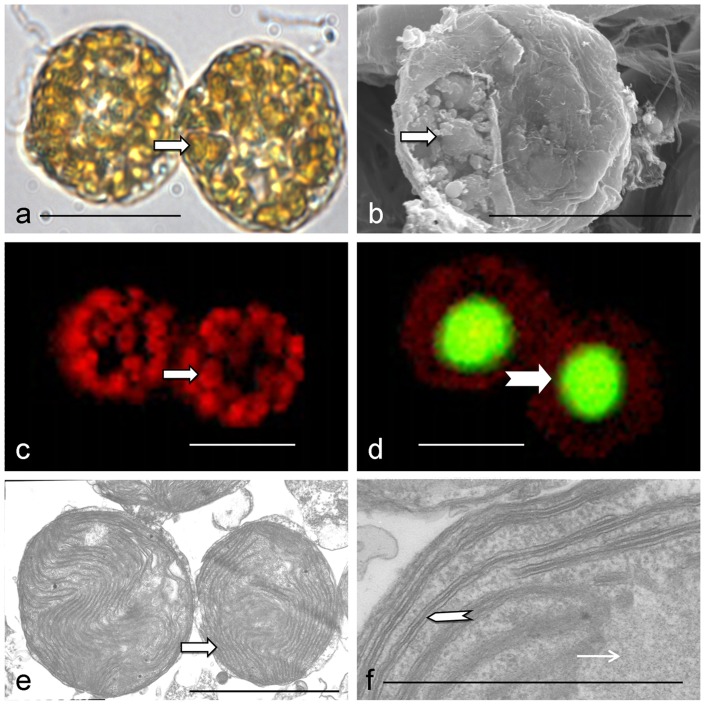
Microscopic characterization of cell colony and cellular features of *P. geminatum* ( = *C. geminatum*). a) Side-view light-microscopic image of a dual-cell colony fixed using the formaldehyde. The cells are packed with chloroplasts of golden-brown color (arrow). b) SEM image of a cell with the round shaped chloroplasts exposed (arrow) due to disrupted cell covering. c) Red fluorescence from Chl. *a* in the chloroplasts (arrow) in a dual-cell colony under blue light excitation. d) Light microscopic image of green fluorescence from SYBR Green I-stained DNA in the nuclei (arrow) in the dual-cell colony superimposed on the red fluorescence from Chl. *a* in the chloroplasts under blue excitation light. e) Transmission electron microscopic image showing two round-shaped chloroplasts (arrow) in a *P. geminatum* cell. f) Ultrastructure of a chloroplast showing thylakoids in stacks of three (thicker arrow) typical of peridinin-containing dinoflagellate chloroplasts and pyrenoid (thin arrow). Scale bars  = 20 µm in (a–d), 3 µm in (e), and 1 µm in (f).

**Figure 4:**
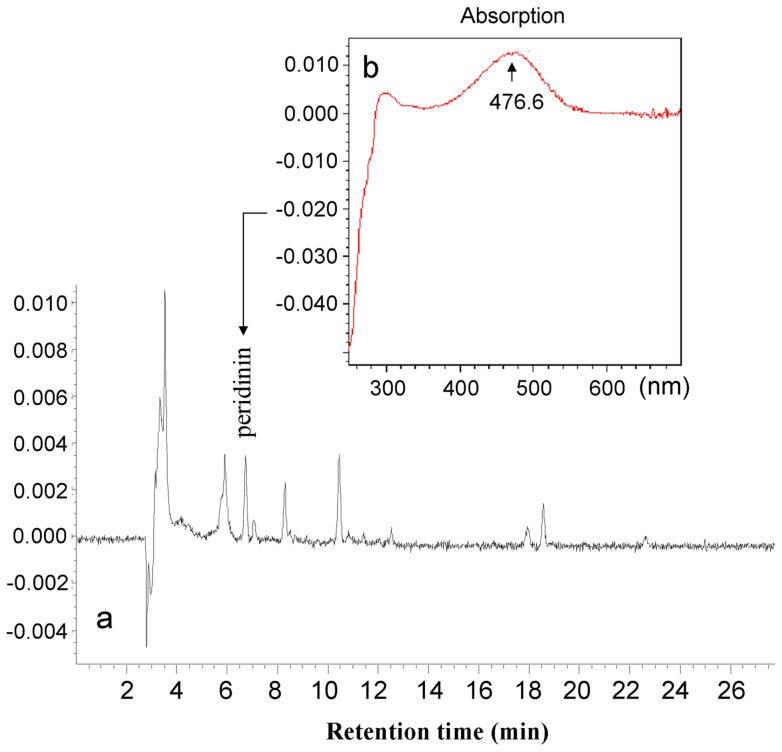
Pigment characteristics of *P. geminatum* ( = *C. geminatum*). a) HPLC chromatogram of the pigments extracted from a water sample during the *P. geminatum* bloom event. b) Absorption spectra of peak 3 shown in (a) (arrow), which was identified as peridinin.

Cells of *C. geminatum* collected during the bloom occurred in pairs (Figure 1a, b; Figure 2e-g; Figure 3a), each were ellipsoidal with size of 20.0–29.9 µm in length and 20.2–30.6 µm in width, giving averaged size of 26.2±2.3 µm (n = 29) in length and 25.1±2.4 µm (n = 37) in width (Figures 2, 4). SEM showed that the two cells were connected through a connection pore (3.2–3.6 μm in diameter) at the attachment point (with small a hinge-like protrusion, Figure 2b, c) which was located near the apical loop of the cell on the bottom and the antapical area of the cell on the top (Figure 2a, c, e–g). Hypotheca exceeded epitheca: the epitheca accounted for 39% of the cell length and the hypotheca 61% on average (Figure 2). Observation of live samples showed that the two flagella moved in concert to enable the two cells to rotate around the longitudinal axis in unison (Figure 2). Examination of fixed samples indicated that in each cell, the cingulum wrapped around the cell body by ∼1.2 turn (Figure 2a), with the first half turn being horizontal and the second half deflecting toward the antapical end of the cell, with a displacement of 38–51% (47% on average, n = 10) of the cell's length (Figure 2). Accordingly, the sulcus turned slightly, with 0.2 turn between the position of the top end and that of the bottom end. The sulcus was deeper than the cingulum, and its top end invaded the epitheca. The apical groove started from the top end of the sulcus and looped anticlockwise on the apical area forming a kidney bean-shaped loop, which was not closed at the end but extended ventrally along side the sulcus.

Under the LM, SEM, and TEM, numerous chloroplasts were observed in the *C. geminatum* cells (Figure 3), occupying most of the intracellular space (Figure 3a, c, d). The chloroplasts were round shaped, 2.3–7.9 µm in diameter, with a mean of 5.2±1.9 µm (n = 25). Under TEM, we observed that the chloroplasts had a pyrenoid and a three-layer envelope (Figure 3f). HPLC analysis showed a peak with retention time and absorption spectrum typical of peridinin (Figure 4) [28]. These results indicate that this species possesses a typical dinoflagellate chloroplast. The oval shaped nucleus is located at the center of the cells (Figure 3d).

### Molecular phylogeny

**Figure 5:**
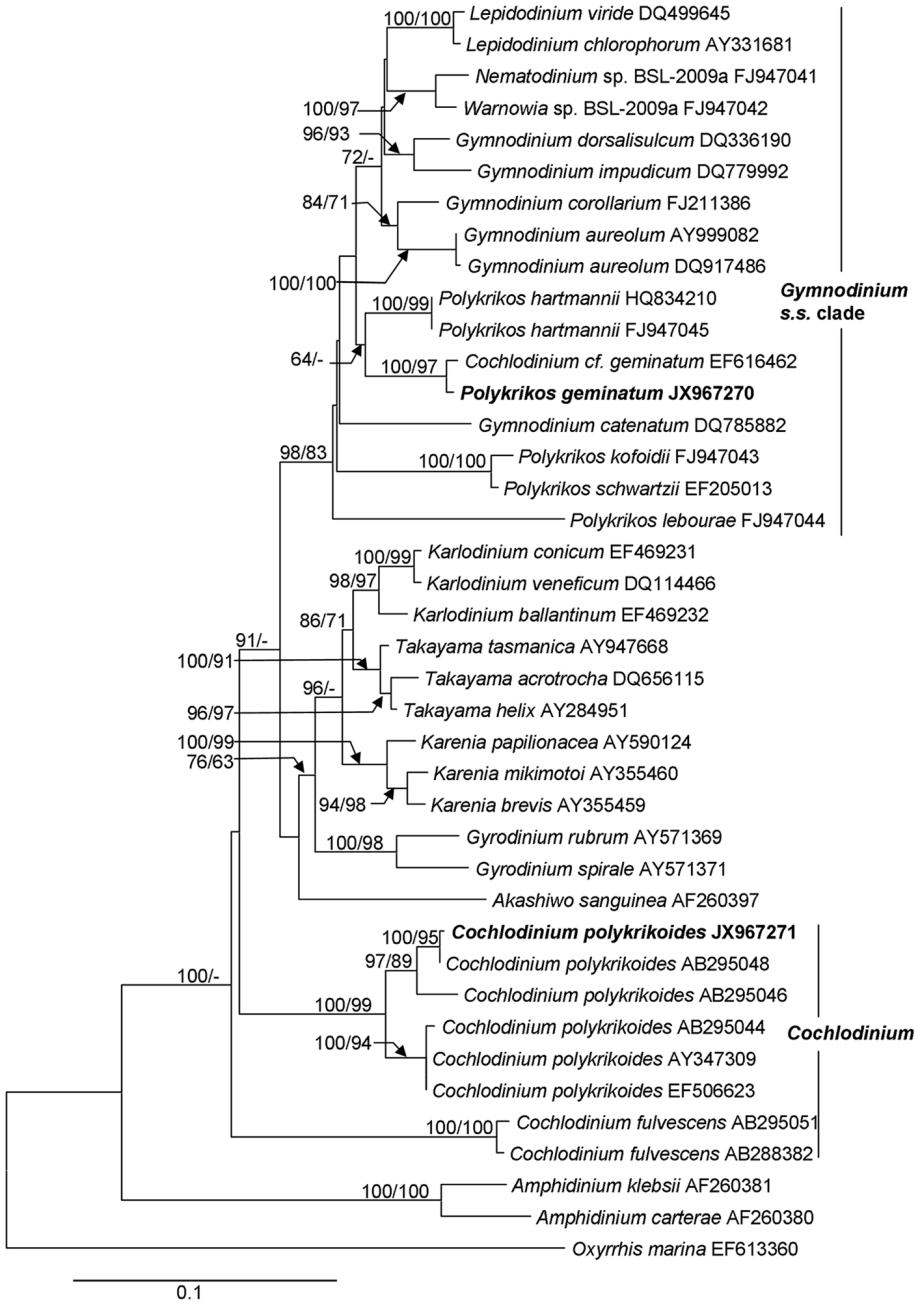
28S rDNA-based phylogeny of *P. geminatum* ( = *C. geminatum*) with other dinoflagellates. Sequences obtained in this study are bold-typed. Support of nodes is based on bootstrap values of ML/NJ with 1000 and 500 resamplings, respectively. Only values greater than 60 are shown. If only one of the two phylogenetic methods yielded significant support, the other is shown with “–”. *Oxyrrhis marina* was used as the outgroup to root the tree. Note that *P. geminatum* is embedded within the *Gymnodinium* s. s. clade with strong support, grouped with *Polykrikos hartmannii*.

**Figure 6:**
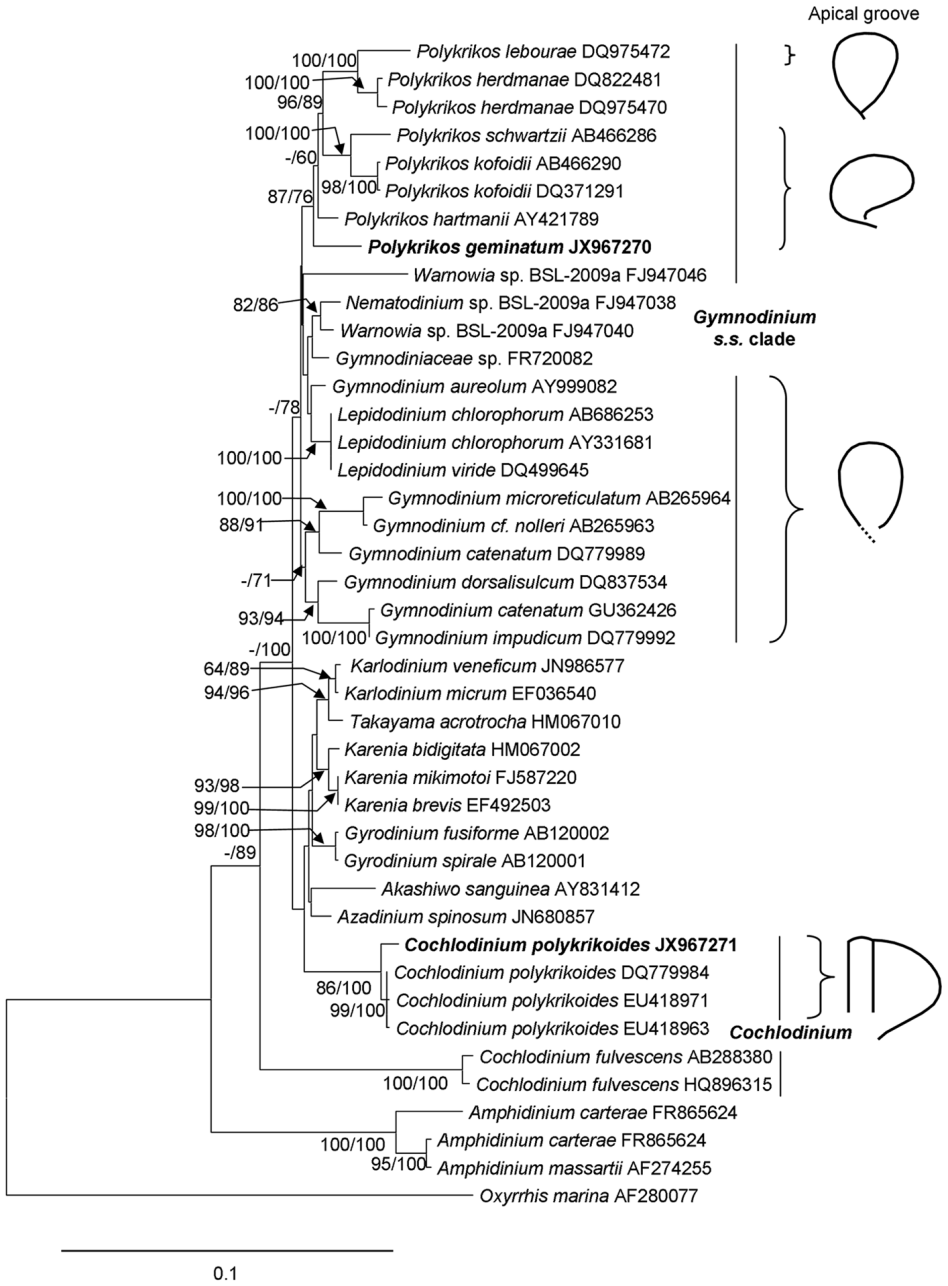
Phylogram of *P. geminatum* ( = *C. geminatum*) with other dinoflagellates inferred from 18S rDNA (left) and drawing of apical groove shapes for major related lineages (right). Sequences obtained in this study are bold-typed. Support of nodes is based on bootstrap values of ML/NJ with 1000 and 500 resamplings, respectively. Only values greater than 60 are shown. If only one of the two phylogenetic methods yielded significant support, the other is shown with “–”. *Oxyrrhis marina* was used as the outgroup to root the tree. Note that *P. geminatum* is affiliated within the *Gymnodinium* s. s. clade, grouped with *Polykrikos* with strong support. From bottom up on the right, *C. polykrikoides* has a U-shaped apical groove; the *Gymnodinium* s. s. clade has three major similar forms of anticlockwise apical grooves (apical views) documented so far: horseshoe-shaped apical groove for *Lepidodinium* spp./*Gymnodinium* spp., anticlockwise kidney bean-shaped open-ended loop for *P. geminatum* /*Polykrikos* spp., and closed horseshoe-shaped apical groove for *P. lebourae*.

**Figure 7:**
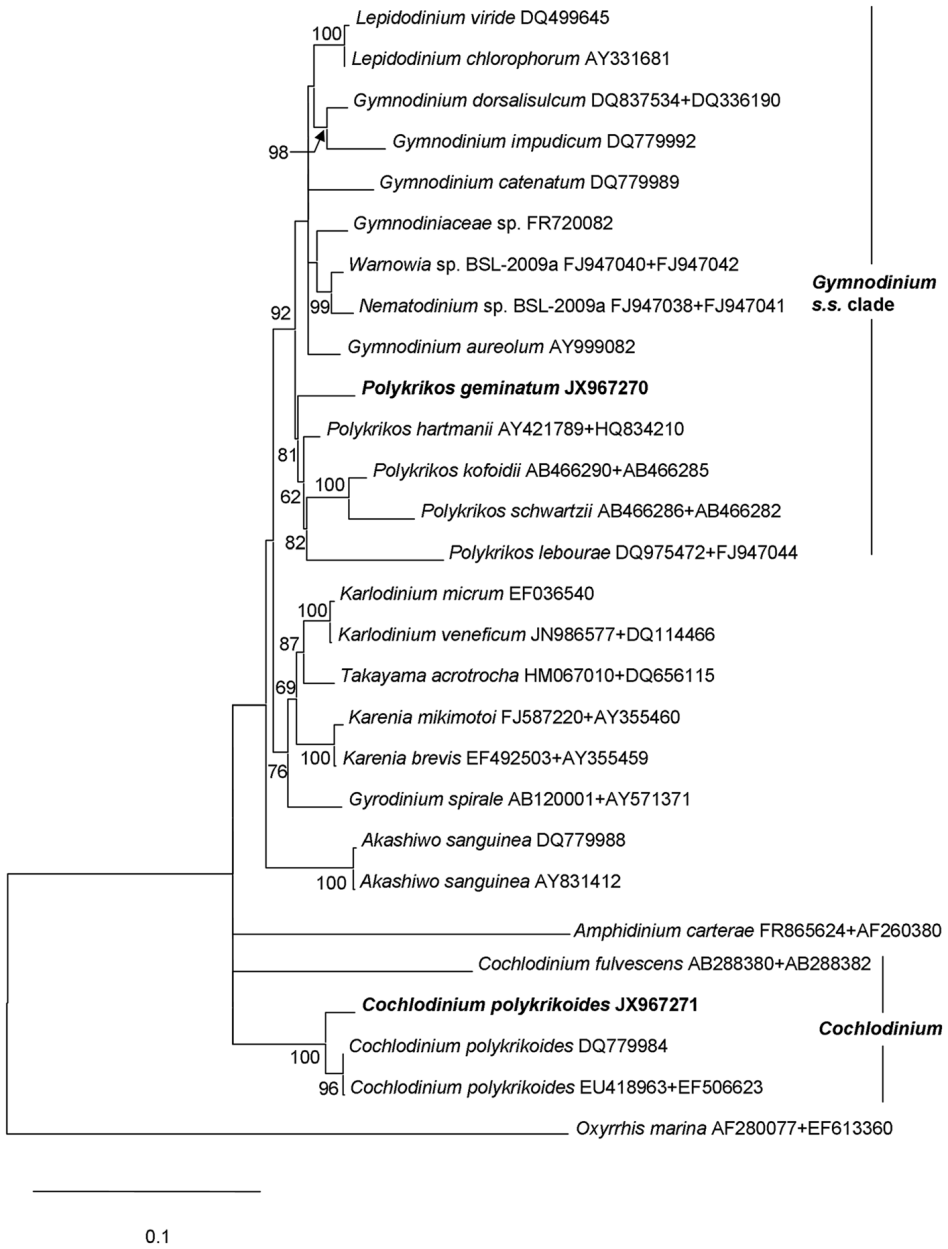
Maximum-likelihood (ML) phylogeny of *P. geminatum* ( = *C. geminatum*) with other dinoflagellates inferred from 18S+28S rDNA concatenated data. Sequences obtained in this study are bold-typed. Support of nodes is based on bootstrap values of ML with 1000 resamplings. Only values greater than 60 are shown. *Oxyrrhis marina* was used as the outgroup to root the tree. Note that *P. geminatum* is grouped with the other species of *Polykrikos* with strong support.

We obtained sequences of a long rDNA fragment from sorted cells of a natural population of *C. geminatum* (3.522 kb; GenBank accession number JX967270) and the culture of *C. polykrikoides* (3.634 kb; JX967271) respectively. These sequences contained partial 18S, ITS1-5.8S-ITS2 (abbreviated as ITS hereafter), and partial 28S (D1–D4). For *C. geminatum* rDNA fragment, the 18S region spanned 1.555 kb, the ITS region 0.703kb, and the 28S region 1.264kb. BLAST analyses showed that *C. geminatum* rDNA sequences were most similar to the counterparts of the *Gymnodinium* sensu stricto (s. s.) clade. Furthermore, the 28 S sequence was almost identical (99%, differing by 10bp out of 0.85 kb) to that of *C. geminatum* isolated from Triabunna, on the East Coast of Tasmania, Australia [15], [29]. In contrast, the sequences obtained from the cultured *C. polykrikoides* were almost identical (99%) to those previously reported for *C. polykrikoides*, confirming correct species identification. However, the sequences from *C. geminatum* and *C. polykrikoides* differed markedly (∼80% identity). Phylogenetic trees based on 18 S, 28 S and 18 S+28 S regions of rDNA were inferred separately, including 38, 40 and 42 (26 species) sequences respectively, representing all known clades of athecate dinoflagellate lineages. For each sequence region, the topologies for Neighbor Joining (NJ) and Maximum Likelihood (ML) trees were similar, consistently placing *C. geminatum* deep within the *Gymnodinium* s. s. clade, in sister relationship with *Polykrikos* with strong bootstrap support. In all these trees, *C. geminatum* was far separated from *Cochlodinium* spp. (including the *C. polykrikoides* strain we analyzed), which formed a distinct clade. In the 28S tree (Figure 5), the Pearl River Estuary *C. geminatum* and the Australian *C. geminatum* were clustered together; these two *C. geminatum* were then grouped with *Polykrikos hartmannii* albeit with weak NJ support. In the 18 S tree (Figure 6), *C. geminatum* was also closest to *P. hartmannii* and other species of *Polykrikos* with moderate NJ and ML supports. Similarly, in the 18 S+28 S ML and NJ trees (Figure 7, Figure S1), *C. geminatum* was placed within the clade of *Polykrikos* with strong bootstrap support, showing a close relationship to *P. hartmannii*, but a far distance from *C. polykrikoides*.

## Discussion

Through a careful examination of morphological, cytological and phylogentic data achieved in this study, we have obtained strong evidence that *C. geminatum* has been misplaced in the genus of *Cochlodinium* and should be moved back to the *Gymnodinium* s.s. clade, specifically to the currently valid genus *Polykrikos*. The result suggests that a combination of apical groove characteristics in combination with molecular phylogenies, instead of cingulum turn number which is currently employed, should be used to discriminate species in the genus *Cochlodinium* from those in *Polykrikos*.

### Morphological evidence: Apical groove versus cingulum turn number

Previous studies have shown that the shape of the apical groove path, which should be viewed from the apical perspective for consistent characterization, appears to be a stable character in unarmored dinoflagellates, such as genera of *Gymnodinium, Gyrodinium, Karenia*, and *Takayama* [1]–[3], [30]. In this study, we confirmed that this character is more stable than the turn number of the cingulum. In some of our samples, depending on how they were fixed and from which angle they were viewed under the light and electron microscopes, cingulum can appear to be between ∼1.10 and 1.25 turns. However, the apical groove exhibited a consistent morphology in all the specimens we examined (Figure 2). In sharp contrast to the apical groove in *C. polykrikoides*, which has a U-shaped trajectory (Figure 6) [31], the apical groove of *C. geminatum* forms an anticlockwise open-ended loop of kidney bean-shape. The presence of an anticlockwise apical groove has been proposed to be a signature character of species in *Gymnodinium* s. s. clade [1]. Consistent with this, the species in *Polykrikos* that were previously assigned to *Gymnodinium* s. s. clade, such as *P. hartmannii* [32] *P. schwartzii* [33], and *P. kofoidii* [1], [34] have similar anticlockwise apical grooves (Figure 6, Table S1). This is despite the fact that within *Gymnodinium* s. s. clade, the shape of groove differs slightly between some lineages. For instance, in *G. impudicum* [35], *G. trapeziforme* [29], *G. catenatum* [36], *G. aureolum* [37], [38], *G. microreticulatum* [39], and *Lepidodinium viride* [40] the apical groove takes an anticlockwise horseshoe shape (Figure 6, Table S1). The apical groove of *P. lebourae* is an anticlockwise nearly closed loop (Figure 6, Table S1) [41]. Whether the subtle difference in the shape of the apical groove represents differentiation at the genus level or species level requires further investigation.

**Figure 8:**
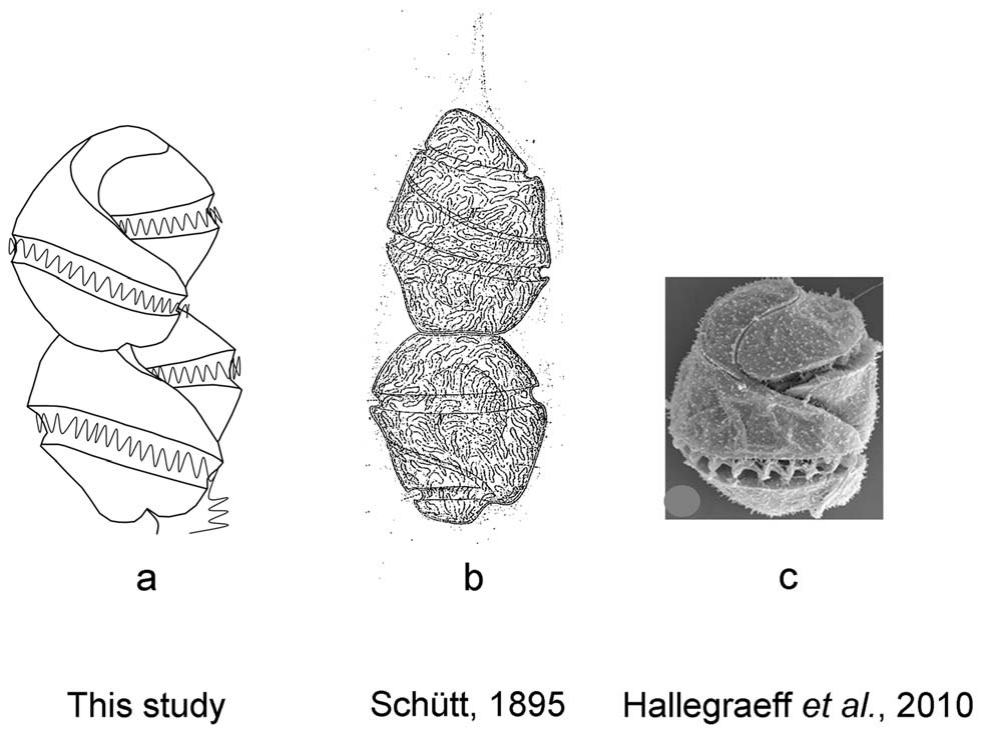
Comparison of historical and current morphological descriptions of the species. (a) Ventral-view line drawing of *P. geminatum* cells showing kidney bean-shaped open-ended loop apical groove from this study. (b) Ventral-view line drawing of *C. geminatum* cells not showing apical groove from Schütt (1895). (c) Ventral-view micrograph of *C. geminatum* cells also showing kidney bean-shaped open-ended loop apical groove from Hallegraeff et al. (2010).

Compared to the apical groove path, cingulum length is less reliable. For example, under SEM, we observed that the cingulum of *C. geminatum* wrapped around the cell body <∼1.5× turns (Figure 2a) while the turn number was hard to estimate accurately under the light microscope. However, the length of *C. geminatum* cingulum was first described as 1.5 or 2.0 turns under the light microscope (Figure 8b; Schütt, 1895, pl.23, Figures 75_2_, 75_3_) [14]. Other than cingulum length, the originally described morphology of *C. geminatum* is very similar to what we observed in the Pearl River Estuary population (Figures 2, 8a, b). Probably the inconsistency was due to confusion between the apical groove and the obliquely turning cingulum because before the 1920's, apical groove ( = acrobase) was not yet documented for unarmored dinoflagellates. The “tail” of the apical groove path obliquely descends toward the sulcus on the ventral side of the cell, which could be misunderstood as the beginning part of the cingulum under the light microscope. As a consequence, the number of cingulum turns might have been overestimated in Schütt (1895) (Figure 8b) [14]. Additionally, sample preservation often alters the morphologies of unarmored dinoflagellates, causing inconsistent estimates of the turn number of cingulum under the light microscope depending on the angle from which the cells are viewed, as noted in the present study.

Some cytological features we observed further support *C. geminatum* to be placed in the *Gymnodinium* s. s. clade, closely related to the genus of *Polykrikos.* The location of the nucleus and many small oval golden-brown (due to peridinin as the major accessory pigment) chloroplasts of *C. geminatum* are consistent with the type description of *P. hartmannii* [32]. Meanwhile, both *C. geminatum* and *P. hartmannii* have been documented as “the connected paired cells ”.

In the *Gymnodinium* s. s. clade, most genera are single-celled while some form pseudocolonies [33], [34]. *Polykrikos* form colonies of 2 to 16 pseudo-cells (zooids) [32]–[34]. In most of *Polykrikos* species, there is no clear separation between neighboring zooids [32]. However, in *C. geminatum,* the two cells are clearly separate cells which are only connected at the attachment point recognizable by the connection pore on each cell (Figure 2). This feature places *C. geminatum* in an “intermediate” state between single-celled and pseudocolony-forming lineages, i.e. probably a basal species in the genus of *Polykrikos*.

### Molecular phylogenetic evidence: nuclear rDNA

Our molecular phylogenetic analyses based on 18 S, 28 S and 18 S+28 S rDNA concatenated sequences have provided definitive evidence that *C. geminatum* falls within the genus of *Polykrikos* (Figures 5, 6, 7, Figure S1) and is only distantly related to the genus *Cochlodinium* (Figures 5, 6, 7, Figure S1). These results place *C. geminatum* deep within the *Gymnodinium s. s.* clade which also includes the genera *Polykrikos*, *Warnowia*, and *Gymnodinium* [10], [32], [41]. In the 18 S phylogenic tree (Figure 6), *C. geminatum* is at the base of the *Polykrikos* cluster, consistent with the basal position posited above based on the cell connection mode. In the 28 S phylogenic tree (Figure 5), our *C. geminatum* strain is allied with the Australian strain also identified as *C. geminatum* (EF616462) and both are grouped with *P. hartmannii*. The lack of other molecular data on the Australian *C. geminatum* strain [15], [29] has precluded further analysis on its relationship with our Pearl River Estuary population; however, its 28 S sequence is almost identical to our *C. geminatum* sequence, which along with morphological resemblance (Figures 2, 8) unequivocally shows that these two populations are the same species. Similarly, in the phylogenetic tree inferred from 18 S+28 S sequences, *C. geminatum* was closely related to *P. hartmannii* and far separated from the genus of *Cochlodinium* with strong NJ and ML support (Figure S1, Figure 7).

In conclusion, *C. geminatum* has cytological features typical of the genus *Polykrikos* (especially to *P. hartmannii*, which is also a photosynthetic species), especially the anticlockwise open-ended loop of kidney bean-shaped apical groove. The affiliation of *C. geminatum* with the genus *Polykrikos* is consistently supported by molecular phylogenies. Therefore, it should be re-classified into the genus *Polykrikos*.

*Polykrikos geminatum* (Schütt) Qiu & Lin.

Basionym: *Gymnodinium geminatum* F. Schütt (1895:165, fig. 75).

Synonym: *Cochlodinium geminatum* (Schütt) Lemmermann (1899:360).

### Re-emphasis of the apical groove – molecular phylogeny approach

The historical taxonomic criteria, based on morphological characters such as cingulum turn number and vertical displacement have been shown not to be always reliable for delimiting genera in Gymnodiniales (including *Cochlodinium*) [1]. In its place, characteristic different shapes of apical grooves have been documented for *Polykrikos, Gymnodinium, Cochlodinium* and other genera in the unarmoured dinoflagellates [1], [2], [13]. In this study, we have confirmed that this apical groove-based distinction is more stable and discriminative when it applies to the case of *P. geminatum*. The combination of this cytological character (supported by other cytological features) with molecular phylogenies has helped unequivocally separate *P. geminatum* from the genus of *Cochlodinium*, with compelling evidence prompt us to transfer it to *Polykrikos*. Based on this case and historical studies, we further propose that classification of species within the genus of *Cochlodinium*, and perhaps all other Gymnodiniales genera, should be based on the shape of the apical groove trajectory (alone or in combination with thecal plate pattern) coupled with molecular phylogeny. This is consistent to what has been practiced for many genera in *Gymnodinium sensu lato* (e.g. [1], [2], [30], [32]).

## Supporting Information

Figure S1 **Neighbor-joining (NJ) phylogeny of** ***P. geminatum*** **( =** ***C. geminatum*** **) with other dinoflagellates inferred from 18S+28S rDNA concatenated data.** Sequence obtained in this study is bold-typed. Support of nodes is based on bootstrap values of NJ with 500 resamplings. Only values greater than 60 are shown. *Oxyrrhis marina* was used as the outgroup to root the tree. Note that *P. geminatum* is closest to *P. hartmannii* but far separated from the genus of *Cochlodinium* with strong support.
(TIF)

Table S1 (DOC)
