# Supplementary figures and images for: Apical Groove Type and Molecular Phylogeny Suggests Reclassification of Cochlodinium geminatum as Polykrikos geminatum
Source: PLoS One. 2013 Aug 19;8(8):e71346. doi: 10.1371/journal.pone.0071346 (PMC3747182; doi:10.1371/journal.pone.0071346)

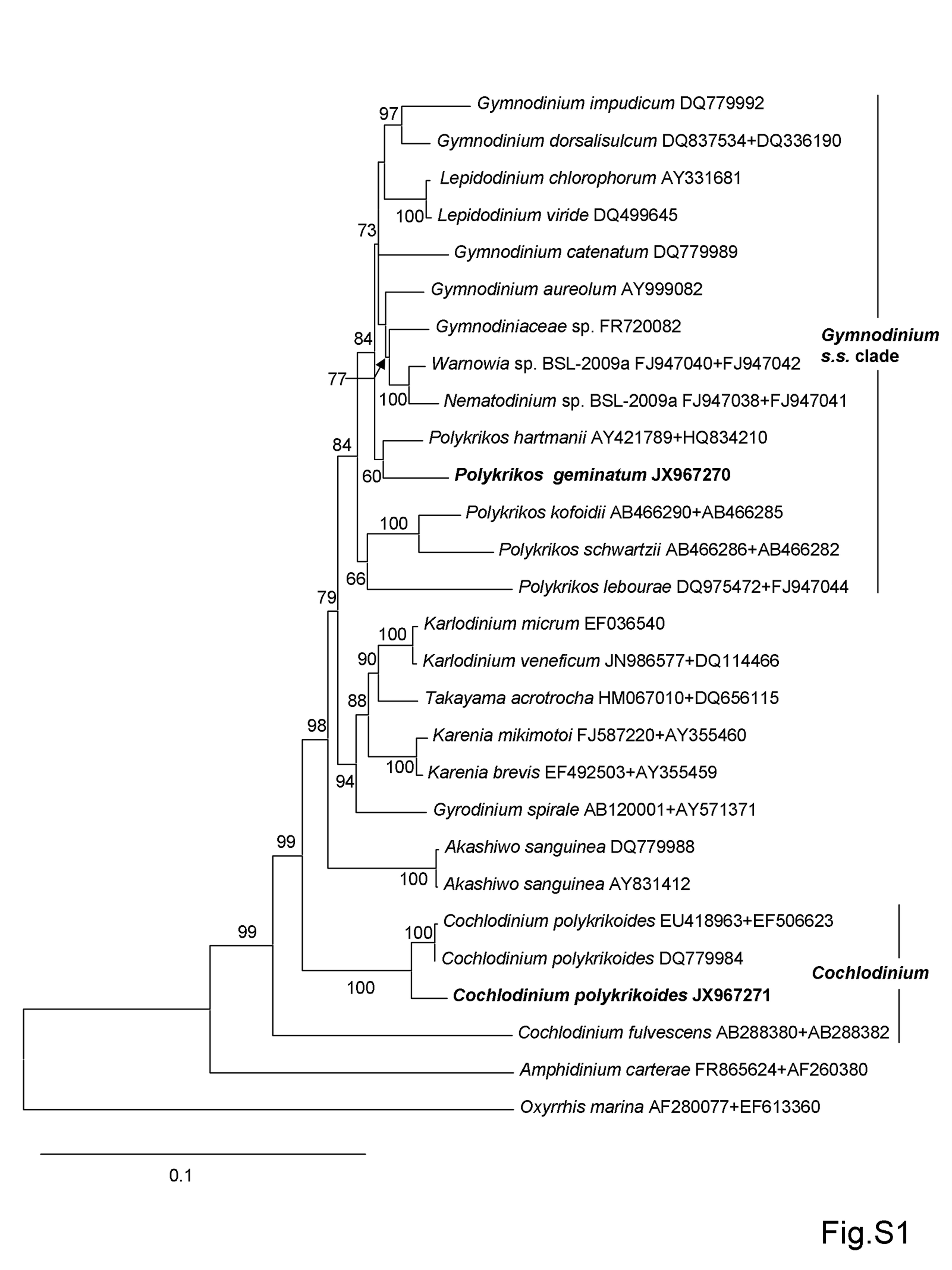

Supplement: Figure S1 — Neighbor-joining (NJ) phylogeny of P. geminatum ( = C. geminatum ) with other dinoflagellates inferred from 18S+28S rDNA concatenated data. Sequence obtained in this study is bold-typed. Support of nodes is based on bootstrap values of NJ with 500 resamplings. Only values greater than 60 are shown. Oxyrrhis marina was used as the outgroup to root the tree. Note that P. geminatum is closest to P. hartmannii but far separated from the genus of Cochlodinium with strong support. (TIF) [file pone.0071346.s001.tif]
