# Supplementary material for: Apical Groove Type and Molecular Phylogeny Suggests Reclassification of Cochlodinium geminatum as Polykrikos geminatum
Source: PLoS One. 2013 Aug 19;8(8):e71346. doi: 10.1371/journal.pone.0071346 (PMC3747182; doi:10.1371/journal.pone.0071346)
Supplement: Table S1 — (DOC) [file pone.0071346.s002.doc]

| TABLE S1. Four types of apical grove images, drawings, and sources in the Gymnodiniales | | | |
| --- | --- | --- | --- |
| **Species** | **Apical groove images** | **Drawings of Apical grooves** | **References** |
| *Gymnodinium impudicum* |  |  | Fraga *et al.*, 1995 |
| *G. aureolum* |  |  | Siano *et al.*, 2009 |
| *G. trapeziforme* |  |  | Attaran-fariman *et al.*, 2007 |
| *G. microreticulatum* |  |  | Iwataki *et al.*, 2006 |
| *G. catenatum* | 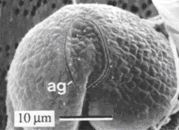 |  | Rees and Hallegraeff, 1991 |
| *Lepidodinium viride* |  |  | Hansen *et al.*, 2007 |
| *Polykrikos schwartzii* |  |  | Matsuoka *et al.*, 2009 |
| *P. kofoidii* |  |  | Matsuoka *et al.*, 2009 |
| *P. hartmanii* |  |  | Hoppenrath *et al.*, 2010 |
| *P. geminatum* | 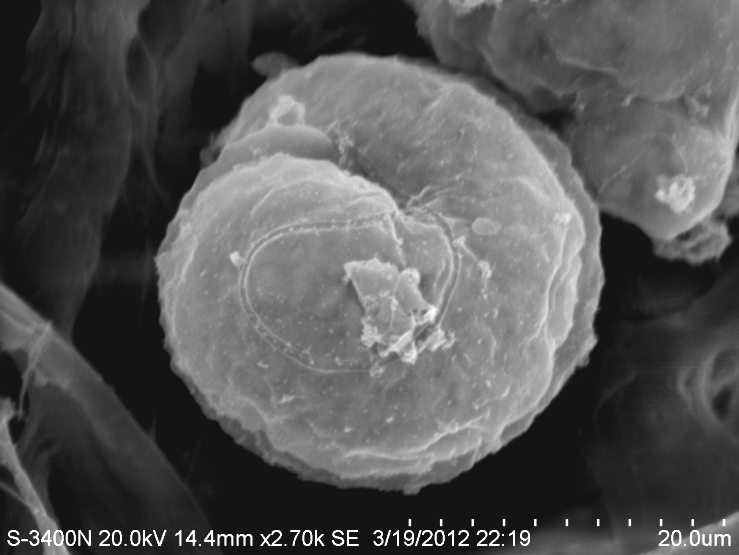 |  | This study |
| *P. lebourae* |  |  | Hoppenrath and Leander, 2007 |
| *Cochlodinium polykrikoides* |  |  | Iwataki *et al.*, 2010 |
